# Navigating conflicting goals and values: a grounded theory exploration of communication skills teachers’ experiences in Swedish medical programs

**DOI:** 10.1007/s10459-025-10464-1

**Published:** 2025-08-22

**Authors:** Emelie Kristoffersson, Hannah Lindgren, Aleksandra McGrath

**Affiliations:** 1 Department of Clinical Sciences, Professional Development, Umeå University, Umeå, 901 87 Sweden; 2 Department of Diagnostics and Intervention (Orthopedics), Umeå University, Umeå, Sweden

**Keywords:** Communication skills, Teachers, Medical students, Grounded theory

## Abstract

The successful teaching of communication skills (CS)—a key competency in medical education—depends on teachers’ expertise. Focusing on the experiences of CS teachers in Sweden, this study aims to explore the challenges they identify in teaching CS and how they adapt their practice accordingly. Individual interviews were conducted with 16 CS teachers, guided by a semi-structured interview guide. Data collection and analysis were iterative, aligning with constructivist Grounded Theory methodology. The teachers experienced a conflict between an idealized view of patient-centered communication taught during CS courses and the actual communication practiced in the clinical context, as well as students’ attitudes. This conflict permeated participants’ perceptions of their teaching goals, the challenges they faced, and the teaching strategies they employed. The teachers’ goals were to convey the purpose of CS, aiming for progress rather than expecting students to reach fixed goals and to promote student ownership in classroom practice. Challenges included students’ negative attitudes towards CS courses, their focus on succeeding rather than developing, and having to teach and examine simultaneously. Strategies focused on supporting learning by transforming negative attitudes towards CS teaching, providing a safe group climate, and offering constructive feedback to reduce defensiveness. This study highlights the need to bridge the gap between idealized CS teaching and clinical practice through institutional support, including curriculum integration, clear assessment frameworks, and recognition of emotional demands on CS teachers.

## Introduction

Communication skills (CS) are widely recognized as a core component of effective medical practice and, as such, are receiving growing emphasis within medical curricula. However, while much research has focused on student experiences (Ruiz-Moral et al., 2019a, b) and outcomes in CS training (Gilligan et al., 2021), the perspectives of the teachers who deliver this training have been relatively overlooked.

While no universal definition is agreed upon (Tan et al., 2021), CS encompasses the ability for a respectful and transparent exchange of information between patients and their caregivers, as well as members of healthcare teams (Makoul, 2001). CS focuses on empathy, clarity, and sensitivity, creating an environment where all parties are comfortable sharing their concerns and perspectives (Tan et al., 2021). Earlier research has demonstrated the benefits of effective CS for both patients and physicians, with correlations between communication abilities and improved patient outcomes, patient satisfaction, compliance to treatment recommendations, decreased rates of medical errors, and reduced physician burnout rates (Boissy et al., 2016; Dwamena et al., 2012; Moore et al., 2013). Reflecting the profession’s commitment to quality improvement and patient-centered care, healthcare providers and institutions now actively champion CS training, with CS recognized as a core clinical competency. Societal standards for physicians’ CS have also evolved alongside the healthcare systems’ recognition of its clinical benefits, with patients increasingly expecting patient-centered interactions with their healthcare providers (Asan et al., 2021; Kalaja, 2023).

Previous research has demonstrated that CS can be successfully taught through dedicated educational interventions (Smith et al., 2007). A recent Cochrane effectiveness review found that CS educational interventions showed positive effects on both overall communication skills and empathy when compared to standard curricula (Gilligan et al., 2021). CS teaching has been shown to achieve behavioral improvement in a simulated environment; however, evidence of its impact on real-world behavior or patient and organizational outcomes remains limited (Gilligan et al., 2021). In modern medical curricula, CS training is often based on a spiraled curriculum approach, with progression from didactic instruction to experiential approaches where subjects and skills are re-explored with greater depth and sophistication as students move through the medical program (Venktaramana et al., 2022). The most common method for teaching CS is through structured courses that incorporate experiential methods, such as role-play, standardized patient encounters, and reflective exercises (Venktaramana et al., 2022).

While effective CS offer numerous advantages, it is essential to acknowledge that teaching the subject may also present specific challenges. Studies show that students experience significant stress, particularly when being observed by peers during small-group sessions with standardized patients and during summative assessments, which may influence their attitudes toward CS teaching (Ruiz-Moral et al., 2019a, b). Furthermore, despite the well-documented value of CS and the effectiveness of CS training, students often hold mixed views on CS courses compared to traditional medical coursework (Berglund et al., 2023).

While research has primarily focused on students, only a few studies have examined this topic from the teachers’ perspective, highlighting both opportunities and barriers in teaching CS (Dewi et al., 2023; Møller et al., 2021). Further exploration of medical teachers’ experiences in teaching CS is important for several reasons. Gaining insight into how they perceive and deliver CS training can help identify gaps and inconsistencies within current medical curricula. Teachers’ experiences influence how effectively students acquire these skills, particularly in light of the challenges students commonly perceive (Ruiz-Moral et al., 2019a, b). Additionally, such exploration can uncover how teachers respond to student difficulties and whether they face unique challenges themselves – factors that must be addressed to support the successful implementation of CS teaching. Ultimately, understanding their experiences can inform faculty development initiatives, thereby ensuring that teachers receive the adequate support they need to deliver high-quality CS education. This study, therefore, aims to explore how CS teachers experience, interpret, and navigate the challenges in teaching CS, as well as the strategies they use to address these challenges.

## Materials and methods

### Study design

This study employed a constructivist grounded theory (GT) approach (Charmaz, 2014), using individual interviews to explore participants’ experiences in depth. Constructivist GT was selected for its dual focus on participants’ perspectives and the researchers’ interpretive understanding of subjective experiences, making it particularly suited to investigate complex phenomena, such as how communication skills teachers navigate their roles and teaching practices. Given the limited prior research on teachers’ experiences in this specific context, this approach provided a flexible yet rigorous framework for developing conceptual understanding directly from the data, thereby avoiding the imposition of predefined categories. We adhered to COREQ reporting guidelines (Tong et al., 2007).

### Setting

This study was conducted in Sweden, where physicians are expected to work in a patient-centered manner, according to an agreement between the Swedish Association of Local Authorities and Regions and the Swedish government in 2015 (*Tillgänglighet och samordning för en mer patientcentrerad vård*, 2015). Patient-centered care is both a foundational value in national healthcare policy (e.g., the Patient Act [2014:821]) and operationalized through structured training, clinical guidelines, and patient-reported experience measures (e.g., in the National Patient Survey). Clinicians are expected to actively involve patients in decision-making, tailor their communication to individual needs, and prioritize the dignity and autonomy of each patient.

Consequently, all medical programs incorporate a patient-centered approach to their CS courses, commonly taught as a part of a Professional development (PD) course. PD is an umbrella for several subjects such as CS, medical ethics, psychology, leadership, and healthcare law. Teachers involved in this subject come from diverse professional backgrounds and frequently straddle the boundary between academic and clinical roles.

CS training follows a longitudinal, vertically integrated, spiral curriculum spanning the entire degree program. While all universities begin with a foundational patient-centered communication model (Larsen & Neighbour, 2014) in preclinical years and progress to more complex scenarios, significant variation exists in the rigor of assessments and attempts at clinical integration. Most programs emphasize formative assessment in small-group settings, but not all progress to summative evaluations – only some incorporate OSCEs or written assessments. Similarly, while didactic training is well-established, clinical integration remains aspirational, with only isolated examples of universities where CS are being assessed during OSCE. This setup creates a fragmented learning experience, with discrepancy between classroom training and clinical application, leaving students without sufficient opportunities to practice CS with real patients under supervision (Berglund et al., 2023).

### Recruitment and participants

Participants were recruited through a national network for PD teachers from all 7 Swedish medical programs using purposeful sampling. Information regarding participation, consent, and study description was sent out with an invitation via email after the study was presented during the network’s annual meeting.

The participants (*n* = 16, eight men and eight women) were teachers employed or associated with medical programs from 6 universities. They both taught and could influence the content of the CS courses, holding positions ranging from regular teachers to course directors. Most participants were, at the time of the interview, actively engaged in clinical practice, with backgrounds in various healthcare professions and specialties, including general medicine, surgery, and psychiatry. None had received any formal CS pedagogical training.

### Data collection

Individual interviews were conducted between 2022 and 2024 via a digital platform (Zoom) (Archibald et al., 2019) by a male medical school graduate and a female political science graduate, both with previous experience teaching CS.

The interviews were guided by a semi-structured interview guide with open-ended questions. The questions explored CS teachers’ backgrounds, teaching objectives and methods, as well as the challenges they encountered while teaching. Follow-up questions were posed in response to the participants’ narratives, asking them to elaborate on their answers.

Interviews lasted between 26 and 77 min and were transcribed ad verbatim.

### Analysis

Data collection and analysis were iterative, per CGT methodology (Charmaz, 2014), with insights gained during the work process used to refine the interview questions. For example, as the interviews proceeded, the participants mentioned a conflict between what is taught in CS courses and the messages students receive during clinical placements. This led us to modify the interview guide to explore different aspects of this conflict. When the data collected reached theoretical saturation, the recruitment was stopped.

The constant comparison technique of GT was used throughout the analysis to explore differences and similarities in the data. First, the authors read and coded the transcripts, with questions such as “What is the participant’s main concern?” posed to the data. Then, the initial codes were compared, discussed, and grouped into preliminary categories that included the content of participants’ experiences. The preliminary categories were compared and refined at joint meetings with all authors, and conceptual relationships between categories were identified and verified in the data, leading to the establishment of a core category. Lastly, all interviews were re-read to ensure that the interpretations were grounded in the data. Additionally, we applied theoretical sensitivity by engaging with the data and existing literature while memoing served as a tool to document emerging insights and refine categories.

Epistemologically, we acknowledge that the data result from an ongoing co-construction between interviewer/researcher and interviewee (Charmaz, 2014). In line with Varpio et al. (Varpio et al., 2017), we also acknowledge that the concept of theoretical saturation is not a neutral methodological step but is shaped by the interpretive lens of the researchers. Our analysis thus emphasized reflexivity and depth of insight over data redundancy. Throughout the data collection, analysis, and writing process, the researchers, therefore, maintained reflexivity by continuously discussing how their roles and assumptions influenced the research process (Verdonk & Abma, 2013). All authors participated in the analysis, challenging each other’s assumptions and mitigating individual biases. The research team consisted of two physicians with varying lengths of experience teaching CS and one political science graduate who was new to teaching communication in medical education. This interdisciplinary composition was helpful when analyzing tensions in the data, such as when teachers expressed internal conflict between teaching idealized communication skills in the classroom while setting different standards during clinical supervision. The political science graduate’s outsider perspective helped question tacit norms that physician-researchers might have taken for granted, while the physicians contributed situated insights into the practical challenges of communication training. Through iterative dialogue, the team critically examined how their differing backgrounds and assumptions shaped interpretations, ensuring that emerging themes were rigorously examined from multiple angles.

## Results

The participants shared their experiences of teaching CS, predominantly in group settings, using experiential learning methods such as peer role-play, interactions with standardized patients, and self- and group reflection.

The participants described self-identified goals for their teaching, highlighted several challenges specific to teaching CS, and shared the strategies they employed to address these challenges, as well as their thoughts on the pedagogical approaches they believed could help mitigate these difficulties.

The analysis resulted in a core category, three categories, and 10 subcategories (see Table 1). Below, these findings are presented and illustrated with participant quotes. Each quote is marked with the participant’s number (1–16) and gender (Man = M, woman = W).

**Table 1:** Summary of the core category, categories and subcategories

A multi-level disconnect: the conflict between idealized CS training and real-world clinical practice	
Categories	Subcategories
Teaching goals shaped by the tension between idealized CS training and real-world clinical practice	-Conveying the importance of CS training to sceptical students -Promoting student ownership in classroom practice -Aiming for progression rather than reaching fixed goals
Challenges in teaching CS exacerbated by the gap between CS training and clinical practice	-Negative attitudes towards CS teaching -Obstacles inherent to group based teaching -The dual role of teacher and examiner -Students focusing on succeeding rather than developing
Strategies for addressing challenges in CS training	-Transforming attitudes through integration and early exposure to clinical practice - Creating a safe group climate in the simulation space -Providing constructive feedback to disarm defensiveness

### Core category: A multi-level disconnect: the conflict between idealized CS training and real-world clinical practice

The core category describes the essence of participants’ accounts, namely the conflicting goals, values, and ideas that existed between the (sometimes idealized) structured, patient-centered communication models taught in dedicated CS courses and the realities of actual clinical practice, as well as students’ attitudes and opinions about CS. This conflict operated at multiple levels.

First, there were divergent perceptions between students and teachers regarding CS, with students often starting the medical program with the perception of CS as innate skills, whereas teachers considered them learnable competencies: “We tell them that you don’t need to be a fully-fledged communicator [yet]. You’re here to practice”(3, M).

Second, teachers recognized that students perceived contrasting communication standards between classroom instruction and clinical practice: “The students are met with double messages […]. And I believe that as a student, you think that I learn one thing at the university and then I learn something else at the clinic” (16, M).

Finally, an internal conflict existed among teachers themselves, whose supervisory approaches varied depending on whether they were teaching theory or guiding clinical practice. Hence, the participants struggled to adhere to the communication standards they taught when supervising students in the clinic: “Unfortunately, when I supervise students in the clinic, I don’t really have time to apply those pieces [consultation methodology] properly” (11, M).

These tensions between theory and practice shaped both the participants’ perceptions of the goals of CS teaching, challenged their teaching practices, and compelled them to adopt their own strategies in response to this conflict.

### Teaching goals shaped by the tension between idealized CS training and real-world clinical practice

Participants articulated their self-identified teaching goals, shaped by the tensions between idealized CS training and the realities of clinical practice, as well as students’ perceptions about CS.

#### Conveying the importance of CS training to sceptical students

Beyond teaching the structure of patient-centered consultations, participants emphasized the need to explicitly anchor its clinical relevance, as students often failed to recognize how CS directly impacts compliance, patient satisfaction, and safety. This pedagogical pressure stemmed from teachers’ observations of students viewing CS as peripheral rather than foundational. Teachers thus felt compelled to justify the CS’s value, transforming their role from mere instructors of technique to advocates for its professional necessity:

“I try to get the student to understand that the goal of focusing on the patient’s part of the consultation is to make an accurate medical assessment, make correct diagnoses, be cost-effective, and increase adherence to treatment. It’s not only about patients seeing us as likable” (15, W).

#### Promoting student ownership in classroom practice

A central goal of CS teaching was to develop a learning environment that fosters self-awareness, reflection, and providing and receiving feedback. Participants underlined that the most effective way of learning was for the students themselves to take as much responsibility as possible. For example, they underlined the importance of allowing students to lead the group while the teacher assumed a withdrawn role, seeing it as more rewarding for the students to receive feedback from their peers than from the teacher: “the best thing is if the students say everything I had intended to say […] because then they have learned something” (6, W).

This ownership model employed experiential learning, mirroring clinical techniques, yet remained contained within pedagogical boundaries. As one participant noted: “When we teach communication, we want to teach you to ask open questions and listen first. It’s simply meta-level. We build the teaching itself on the same building blocks as the consultation with the patient” (10, M). While this approach developed classroom competence through peer interaction, the parallel between training and clinical practice remained implicit rather than examined. The emphasis on student leadership and peer feedback cultivated engagement but left unaddressed how these idealized interactions might require adaptation for clinical realities.

#### Aiming for progression rather than reaching fixed goals

Another important goal was to focus on progression and self-awareness based on the individual resources possessed by students, rather than achieving a fixed set of skills. Furthermore, teachers reported two interrelated goals: challenging students’ assumption about CS as innate traits and counteracting these assumptions by emphasizing the learnable nature of CS: ”You are not born a perfect communicator; [CS] can be developed, and it may take time” (9, M).

The participants believed that although students had differing levels of motivation, every student had the potential to improve and progress and that it was their role to guide them toward this improvement to reach a solid basic level of CS proficiency: “The teaching aims to raise the minimum level properly” (8, W).

### Challenges in teaching CS exacerbated by the gap between idealized CS training and real-world clinical practice

Participants identified several challenges stemming from the disconnect between idealized CS training and clinical practice as well as students’ attitudes.

#### Negative attitudes towards CS teaching

An important challenge for the participants was that students had negative perceptions of CS teaching and patient-centredness, characterizing them as “vague”, “time-consuming”, and “overly theoretical”. Trying to engage skeptical students was exhausting: “It is challenging, like a difficult patient meeting” (11, M).

The participants believed that the source of students’ skepticism was that they arrived at the medical program with pre-existing ideas about the physicians’ role and that CS courses started during the pre-clinical years, i.e., before the students had ever met a patient. Students, in turn, observed similar negative attitudes among clinical supervisors:

“We teach certain things, and then students encounter hundreds of clinical supervisors who all act very differently. Sometimes there isn’t direct alignment between what we teach and how supervision happens—it can even become contradictory at times” (9, M).

Despite frustration caused by negative attitudes from clinical supervisors, the CS teachers acknowledged the difficulties clinical tutors face when trying to use patient-centered communication, seeing how the content of CS courses is not easily transferable to clinical reality:

“It is not good as it is now because it is not adapted to reality. It’s good not to use leading questions, but at the same time, you have to control it [the consultation] a little if you have only 15 minutes with the patient. Sometimes, it gets slightly ridiculous if you look at it from a clinical perspective” (4, M).

#### Obstacles inherent to group-based teaching

The nature of CS teaching, occurring in group settings, presented a challenge as negative group dynamics could hamper the students’ development. For students, a deep-seated fear of being humiliated in front of their peers also constituted a barrier to learning:

“Many students are nervous about this situation, where everyone watches you, about being scrutinized and making mistakes or saying something stupid”(15, W).

Furthermore, large groups made it difficult to give students individual feedback:

“It’s common to get insufficient feedback, and we have feedback in a group; it’s not optimal, especially if it’s sensitive. Also, we have limited time and resources” (10, M).

#### The dual role of teacher and examiner

Teachers also encountered difficulties in assessing the students. The teachers’ focus on students’ individual progression rather than meeting a set of pre-defined criteria and vague/non-specific learning objectives resulted in teachers rarely failing students:

“We have learning objectives for each semester, but truth be told, almost all students fulfill those objectives. And it’s not our ambition to exclude students for making a poor performance. We try to see to their ability in the long run” (3, M).

The participants expressed concerns that their formative assessment of students’ CS risked being subjective. Teaching and assessing, which took place simultaneously, generated uncertainty:

“It is a problem or an advantage, depending on how you look at it, that you often teach and examine simultaneously. You are assessed at the same time as you train. There is a gray area between teaching and examination” (13, W).

#### Students focusing on succeeding rather than developing

Another recurring challenge for teachers was that students often focused on succeeding rather than developing and thus perceived constructive feedback as a personal failure, which created an emotional burden for teachers. Participants believed that since personal qualities are an essential part of communication, this made students susceptible to criticism, which made it challenging for them to provide constructive feedback, as they feared causing discomfort.

“Most students are those type A personalities who want to perform on top, even though they are [early in their medical education]. Many students have very high ambitions and want to know everything at once. And when they are told something is wrong, they can feel defeated, and you must deal with that kind of reaction” (3, M).

### Strategies for addressing challenges in CS training

To facilitate meaningful learning, participants implemented a range of adaptive strategies. While some approaches drew on conventional pedagogical methods, others were specifically applied to address the theory-practice dissonance students encountered.

#### Transforming attitudes through integration and early exposure to clinical practice

Referring to the fixed ideas about the physician’s role among new medical students, participants teaching in preclinical semesters described how they had to work with changing these attitudes to CS in parallel with teaching. Early clinical exposure was seen as crucial to increase motivation to learn CS: ”They need to recognize the need. […] The students might need more clinic early on.” (6, W).To change students’ and clinical supervisors’ attitudes and increase students’ motivation to learn CS, the participants also saw a need to clarify the learning objectives and integrate CS into clinical subjects:

”You may need to work in both directions. To get [CS teaching] to work more with assessment instruments, there should be clear criteria for what is considered approved and what is not. But also to work with the clinical subjects and make them more holistic and integrate CS aspects there” (6, W).

CS teachers who could bridge the gap between the clinic and PD/CS constituted another solution to change the negative attitudes: ”Integration with the clinic is essential” (4, M).

#### Creating a safe group climate in the simulation space

To address the tensions, reduce the fear of humiliation, and encourage student engagement, participants prioritized building trust and creating a safe, non-judgmental group climate. Strategies included letting the students formulate a code of conduct, in which it was agreed that thoughts and feelings that were shared would not be disclosed to others outside the group, validating the students, and using humor to take the edge of embarrassing situations:

“You have to create safe space and have humor; you have to laugh at yourself when things go badly. You can’t take yourself so seriously, but you can laugh and say it’s good that we practice” (8, W).

Furthermore, the participants underlined the importance of prompting the student to share any apprehension to normalize that it is common to feel nervous when training CS: ”By verbalizing it, you might be able to ease the anxiety and perhaps get advice on how to handle it.” (10, M).

#### Providing constructive feedback to disarm defensiveness

To reduce the risk of students feeling scrutinized and criticized, participants emphasized the importance of well-formulated and constructive feedback, avoiding overtly critical feedback. An important aspect was to distinguish between personal characteristics and CS and directing feedback towards tangible actions, starting with positive reinforcement, followed by a constructive component, and clarifying that there were alternative strategies for addressing a difficulty:

“You are also responsible for highlighting the positive and describing why it is positive. When I give my criticism, I can say: “On a good day, I might have done it like this”. I think that the structure [of giving feedback] that we use is not judgmental but rather invites us to discuss that there are different ways of doing things” (15, W).

## Discussion

This study examines the experiences of CS teachers in Swedish medical schools, providing insights into how they make sense of, adapt to and manage the complexities of teaching CS in both classroom and clinical settings. The tension between idealized CS teaching and clinical realities, as well as students’ perceptions about CS, shaped how teachers framed their goals, encountered obstacles, and developed pedagogical responses. Supported by formal learning objectives, the participants constructed their goals for teaching: conveying the value of CS, fostering students’ self-awareness, and encouraging continuous progress rather than achieving fixed skill levels. However, these goals were frequently obstructed by negative attitudes towards CS among students and faculty as well as the prevailing mindset of students who prioritized success over progression. Teachers navigated this tension by fostering safe and reflective learning spaces, transforming attitudes towards CS, and striving to bridge the divide between CS education and clinical practice.

### Navigating conflicting goals and values

Efficient communication is a cornerstone of medical practice. Nevertheless, despite its importance, the participants in this study found themselves having to justify the relevance of CS to students, highlighting a contrast with more traditional subjects, such as anatomy or histology, where the benefits are typically unquestioned. This persistent need to “prove” the value of CS education against prejudices has also been noted as a barrier to the introduction of CS training in Spain (Ruiz Moral et al., 2020). Furthermore, our participants reflected on the internal conflict they experienced as teachers between teaching CS and managing the realities of clinical practice. Previous studies have also found that CS skills that teachers promote in the classroom do not easily transfer into the reality of clinical practice (Møller et al., 2021). A previous study among CS teachers and their educational leaders in clinical departments has also shown the gap that exists between structured learning and real-world clinical practice (Dewi et al., 2023). Similarly, none of the clinical leaders utilized the teachers’ competence in teaching CS, and some even perceived it as unimportant (Møller et al., 2021).

The tensions and conflicts described by our participants reflect the contradictions between the university-based CS training environment and the realities of clinical practice. While the former emphasizes idealized, structured, patient-centered communication, the latter is shaped by time constraints, efficiency demands, and different professional norms (Dewi et al., 2023; Elmberger et al., 2019). Participants’ experiences illustrate how these competing values create conflicting messages for students and internal dilemmas for teachers—particularly when their own behavior in clinical supervision diverged from the communication standards they promoted in formal teaching: while courses emphasized an idealized consultation approach, clinical supervision often required them to adapt to the constraints of real-world healthcare. This duality highlights an emotional burden inherent in CS teaching (Dewi et al., 2024).

Another recurring theme in participants’ accounts was the sensitivity surrounding feedback on CS. Unlike feedback in more technical domains, CS feedback often touches on personal characteristics, making it particularly challenging for students who are used to excelling academically but may be less comfortable exploring their weaker sides (Ruiz-Moral et al., 2019a, b). This challenge was further compounded by students’ tendency to focus on performance rather than development, a mindset that can hinder their ability to engage with complex, unpredictable patient consultations (Knights & Kennedy, 2006). This combination of sensitivity and a performance-focused mindset not only hinders for learning but also places significant emotional and pedagogical demands on teachers. It seems their constructive but cautious feedback on CS was motivated primarily by fostering student learning while simultaneously balancing the emotional labor inherent to their role.

Participants also highlighted a tension between promoting students’ progress and ensuring they meet established criteria. In other subjects in the curriculum, students are expected to meet minimum standards to progress in their education; yet, in CS, the teachers emphasized improvement over achieving fixed benchmarks. The perspective of CS teachers aligns with research focusing on the broader challenge of navigating clinical uncertainty, where exposure and reflective practice help students gradually build tolerance for ambiguity (Moulder et al., 2023; Stephens et al., 2024). However, this inconsistency in how CS are integrated and assessed within the broader medical curriculum mirrors findings where students’ performance aligns poorly between learning environments (Dewi et al., 2024; Laidlaw & Hart, 2011; Rees et al., 2002).

Furthermore, assessment itself posed a significant challenge for participants. Many described difficulties in simultaneously teaching and evaluating students, particularly when learning objectives were perceived as fluid or ambiguous. This dual role contributed to uncertainty about how best to measure students’ progress while maintaining a supportive teaching approach. Similar tensions have been observed in studies exploring the teaching of professionalism and have been found to lead to ‘assessment avoidance’ (Liljeholm Bång et al., 2024). Our findings suggest assessment avoidance persists due to teachers’ beliefs in natural skill progression during CS and clinical training and the lack of clear learning objectives for CS. This implies that current formative approaches alone may be insufficient without faculty development and clearer competency frameworks.

### Implications for medical education

The challenges faced by CS teachers, such as the clash between idealized CS teaching and clinical realities, negative attitudes toward CS among students and staff, and students’ sensitivity to feedback increased participants’ workload. Improving CS education and the conditions for those teaching requires reconciling the contradictions between educational ideals and clinical realities. This involves addressing cultural barriers, both in the classroom with students and for the faculty, elevating the status of CS within the curriculum, and fostering positive perceptions of learning. Efforts to reshape attitudes should begin early and run parallel to early clinical exposure to provide context for CS training and bridge the perceived gap between theory and practice. Students also need support in understanding that learning CS is a gradual process, where mistakes are both inevitable and valuable, as CS are acquired skills, not an innate trait. More precise learning objectives for CS are needed to support teachers in conducting consistent assessments. Finally, faculty development initiatives should support teachers, equipping them to manage emotional labor and navigate institutional tensions.

### Methodological considerations

Our choice of method resulted in a heterogeneous group of participants and nuanced interview material. However, the study has some limitations to consider. First, the participants may not represent all teachers in CS education; e.g., those who face more challenges in teaching CS may be more inclined to participate. However, the varied experiences shared by the interviewees underscore our study’s broad enrollment and credibility. Second, participants may have withheld sensitive information due to concerns about students’ understanding. However, the openness of the responses indicates that participants felt comfortable sharing their experiences. Finally, the study is limited to data from a single country; nevertheless, our participants’ experiences parallel descriptions from other contexts (Ruiz Moral et al., 2020). Therefore, we believe the results are transferable to and relevant to other Euro–American contexts.

## Conclusions

This study underscores the challenges of teaching CS in medical education. Addressing barriers to CS education requires addressing the gap between idealized CS teaching and clinical practice through institutional support. This includes re-evaluating its perceived value, integrating it into the medical curriculum, and continuing work on developing clear assessment frameworks. Leadership should also recognize the emotional demands on CS teachers. Such efforts are essential to the effectiveness of CS training in preparing future physicians.

## Data Availability

No datasets were generated or analysed during the current study.
